# Prevalence and factors associated with general anxiety symptoms severity among older adults registered with the Primary Healthcare Corporation during the COVID-19 pandemic: A cross-sectional study

**DOI:** 10.5339/qmj.2023.17

**Published:** 2023-08-08

**Authors:** Raana Nishat Syed, Aza Ziyada, Hena Athar, Javaria Khan, Mujahed Shraim

**Affiliations:** ^1^Primary Healthcare Corporation (PHCC), Doha, Qatar; ^2^Public Health Department, College of Health Sciences, QU Health, Qatar University, Doha, Qatar. E-mail: mshraim@qu.edu.qa ORCID: 0000-0001-7972-8210

**Keywords:** COVID-19, SARS-CoV-2, Anxiety, GAD-7, Older adults, Cross-sectional study

## Abstract

Background: Primary care-based studies examining the prevalence of anxiety symptoms severity and associated factors among older adults during the COVID-19 pandemic are scarce. The study aims to determine the prevalence of general anxiety symptoms severity and associated sociodemographic and physical health characteristics, including SARS-CoV-2 infection history, among older adults in primary care in Qatar during the COVID-19 pandemic.

Methods: A cross-sectional study was conducted using a random sample of older adults aged 60 years and above (n = 337) from all primary health care centers (n = 28) of Qatar’s Primary Health Care Corporation. Participants were interviewed via telephone by family physicians between June and August 2020. General anxiety symptoms severity was assessed using the Generalized Anxiety Disorder 7-item Scale (GAD-7). Descriptive statistics and ordinal regression were used to analyse the data.

Results: The mean age of participants was 65 years (ranging from 60 to 89 years), standard deviation = 4.8. About 49.0% and 32.0% of participants were females and of Qatari nationality, respectively. The prevalence of minimal, mild, moderate, and severe general anxiety symptoms was 82.5%, 13.9%, 3.0%, and 0.6%, respectively. Around 33.5%, 63.5%, and 3.0% of participants had unknown, negative, or positive SARS-CoV-2 infection histories, respectively. Females had greater odds of higher levels of anxiety symptoms severity (odds ratio (OR) 2.34; 95% confidence interval (CI) 1.22, 4.50; p = 0.011). As compared to participants with unknown SARS-CoV-2 infection status, those with a negative and positive SARS-CoV-2 infection history had increased odds of higher levels of general anxiety symptoms severity by 2.48 (95% CI 1.17, 5.24; p = 0.017) and 7.21 (95% CI 1.67, 31.25; p = 0.008), respectively. Age, marital status, living arrangements, nationality, and the number of medical conditions had no statistically significant associations with general anxiety symptoms severity.

Conclusions: Most older adults experience minimal to mild anxiety symptoms during the COVID-19 pandemic. Female gender and confirmed or suspected SARS-CoV-2 infection history are independent predictors of more severe anxiety symptoms among older adults.

## Introduction

As of 7 January 2023, severe acute respiratory syndrome coronavirus 2 (SARS-CoV-2) causing coronavirus disease (COVID-19) led to more than 657.9 million confirmed cases and more than 6.6 million confirmed deaths globally.^1^ Older adults and those with pre-existing medical conditions were identified as being at increased risk of severe complications and mortality from COVID-19.^2^ To prevent the spread of SARS-CoV-2, most countries implemented drastic preventative measures, such as physical distancing and lockdowns. These measures and fear of COVID-19 had a profound negative impact on the psychological, social, and economic well-being of the general population worldwide^3,4^ Therefore; mental health was identified as a global research priority to better understand and mitigate the negative impact of the COVID-19 pandemic on the psychological well-being of the community at large, especially among older adults and individuals with underlying medical conditions who are at higher risk of COVID-19-related complications and death.^5–7^

Few systematic reviews have examined the impact of the COVID-19 pandemic on mental health among older adults and individuals with underlying health conditions. One systematic review conducted in March 2021 showed that older adults reported significantly lower depression, anxiety, and stress symptoms than young and middle-aged adults during the COVID-19 pandemic.^8^ However, the same systematic review found that patients with chronic physical conditions had significantly higher levels of depression and stress symptoms severity than healthy subjects. Another systematic review conducted in May 2020 reported an increased prevalence of anxiety and depression symptoms severity in the general population during the early phase of the COVID-19 pandemic compared to pre-COVID-19 pandemic data. Still, no significant differences were observed among patients with underlying health conditions or healthcare workers.^9^ This systematic review also identified some common protective factors (older age and good economic and education levels) and risk factors (pre-existing mental disorders, female gender, and concerns about contracting SARS-CoV-2) for mental health outcomes among the general population, patients with pre-existing conditions, and healthcare workers.

Previous population-based studies suggest that the severity of anxiety symptoms varies according to demographic and health characteristics, including SARS-CoV-2 infection status. The magnitude and extent of the negative impact of the COVID-19 pandemic on mental health are likely to vary between countries and population subgroups according to various contextual factors, such as the severity of the pandemic, pandemic preparedness, local public health information, mental health and social care services, socioeconomic status, and cultural and spiritual practices.^10–14^ Most previous studies examining mental health outcomes among older adults and patients with underlying medical conditions during the COVID-19 pandemic were conducted in China, Europe, and North America using general population samples and self-reported data, with very few studies from the East Mediterranean Region, particularly from the Arabian Gulf Region.^9,15,16^ In addition, primary care-based studies examining the prevalence of anxiety symptoms severity and associated factors during the COVID-19 pandemic are scarce. Assessment of the psychological well-being of vulnerable populations, such as older adults with a high prevalence of underlying physical conditions during the COVID-19 pandemic, has important implications for identifying and targeting high-risk groups for COVID-19-related adverse mental health outcomes with tailored psychological support interventions. Therefore, this study aimed to determine the prevalence of general anxiety symptoms severity and associated sociodemographic and physical health characteristics, including SARS-CoV-2 infection history, among older adults in primary care in Qatar during the COVID-19 pandemic.

## Materials and Methods

### Study design, setting, and population

This was a cross-sectional study of older adults aged 60 years and above registered with any Primary Health Care Corporation (PHCC) centre in Qatar between June and August 2020. The PHCC is the leading provider of publicly funded primary healthcare services in Qatar, including twenty-eight centers distributed nationwide.^17^ The PHCC uses a universal electronic medical records (EMR) system across all the PHCC centres. All older adults aged ≥60 without underlying mental health conditions were eligible for inclusion in the study. All participants gave verbal informed consent to participate in the study. The Institutional Review Board of the PHCC research subcommittee approved the study (approval number PHCC/DCR/2020/07/072). All participants with moderate or severe general anxiety symptoms severity were offered a consultation with a general physician.

### Sampling and recruitment

In 2012, the prevalence of mild to severe generalized anxiety disorder among the PHCC population aged 50 to 64 years and ≥65 years were estimated at 14.5% and 13.6%, respectively.^18^ Based on this, a sample size of 306 older adults aged ≥60 years was needed to address the aim of the current study, with an estimated prevalence of moderate to severe general anxiety symptoms severity at 15%, with a 4% margin of error rate and a 95% confidence level. However, we increased the sample size by 10% and included 337 individuals to account for any missing data or withdrawal from the study.

**Figure 1.**
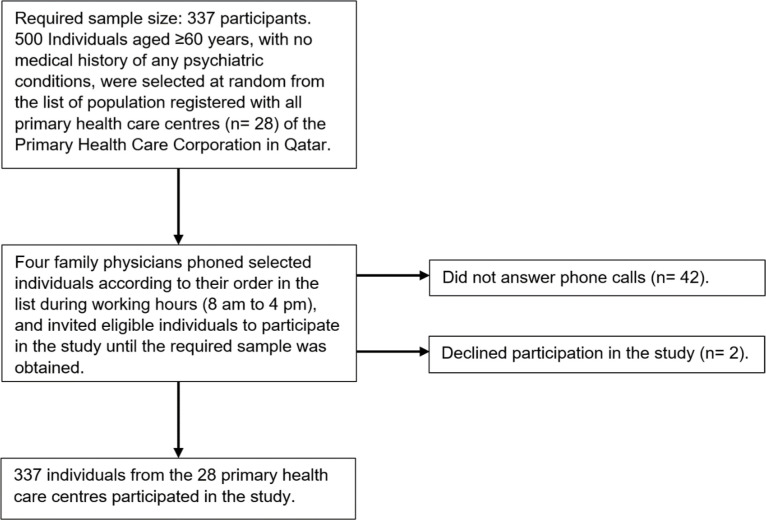
Sample selection and recruitment flow chart

The Health Information Management department of the PHCC selected a list of eligible individuals (n = 500) from the twenty-eight PHCC centres using a simple random method. The PHCC’s unique identifier numbers and phone numbers of the selected individuals were shared with the research team of the current study. Four family physicians phoned eligible participants during working hours (8 am to 4 pm) and invited them to participate in the study according to their random order in the list. Individuals who did not answer the first phone call were phoned again twice at various times during the study period. Participants were enrolled in the study until the required sample size was reached (n = 337). Overall, forty-two individuals did not answer the phone calls, and two declined participation in the study (Figure 1).

### Data collection

Study participants were interviewed in Arabic or English (based on their preference) by one of four interviewing family physicians using a standardized questionnaire script. Data on marital status (married, not married (including single, divorced, widowed)), living arrangements (lives with family, lives alone), and general anxiety symptoms severity were collected. The Generalized Anxiety Disorder 7-item Scale (GAD-7) was used to measure the prevalence and severity of general anxiety symptoms among participants (Supplemental file 1). The GAD-7 has a sensitivity of 89% and a specificity of 82% for GAD.^19^ The GAD-7 scale measures the frequency of experiencing anxiety symptoms in the last two weeks using a 4-point Likert scale (0= “not at all,” 1= “several days,” 2= “more than half the days,” and 3= “nearly every day”). The overall GAD-7 score ranges between 0 and 21, and higher scores indicate higher severity of anxiety symptoms (1-4 = minimal symptoms, 5-9 = mild symptoms, 10-14 = moderate symptoms, and 15-21 = severe symptoms).^19^ We used the officially translated GAD-7 screening tool in Arabic, which was translated by the Mapi Research Institute using an internationally accepted translation methodology and is freely available from the Patient Health Questionnaire (PHQ) Screeners website (www.phqscreeners.com).

EMRs were used to extract data on the following variables: age, gender, nationality (Qatari, other), previous SARS-CoV-2 infection history (unknown (not tested), negative, positive), and medical history (yes, no) of any of the following medical conditions (diabetes, hypertension, cardiovascular disease, cerebrovascular disease, dyslipidaemia, asthma or chronic obstructive pulmonary disease, liver disease, chronic kidney disease, and cancer). Several medical conditions (comorbidities) were categorized as 0, 1, 2, 3, and ≥ 4.

### Statistical analysis

Descriptive statistics were used to summarise the data. Continuous variables were summarised using mean and standard deviation (SD). Categorical variables were summarised using frequencies and percentages. Univariable and multivariable ordinal regression analyses were used to assess the relationship between participants’ sociodemographic and health characteristics and general anxiety symptoms severity. The odds ratio (OR) and associated 95% confidence interval were used to measure association. Associations with a p-value of ≤0.05 were considered statistically significant. All statistical analyses were performed using STATA (versions 16).^20^

## Results

### Characteristics of participants

The sociodemographic and health characteristics of participants are summarised in Table 1. 337 persons from the 28 PHCC centres participated in the study. The mean age of participants was 65 years (ranging from 60 to 89 years), and 49.0% were females. About 32.0% and 89.3% of participants were Qatari and living with families, respectively. Most participants (92.9%) were married. Around 33.5%, 63.5%, and 3.0% of participants had unknown, negative, or positive SARS-CoV-2 infection history, respectively. About 19.3% of participants had no history of chronic physical conditions. Approximately 6.6%, 21.1%, 26.1%, and 16.9% of participants had one, two, three, and four or more physical conditions, respectively. The most common conditions were hypertension (59.0%) and diabetes (57.3%), and the least common conditions were cerebrovascular disease and liver disease (1.8% each).

### Prevalence of general anxiety symptoms severity

The mean GAD-7 score was 1.8 (SD=3.0), and the median GAD-7 score was 0 (IQR=3). The prevalence of minimal, mild, moderate, and severe anxiety symptoms was 82.5%, 13.9%, 3.0%, and 0.6%, respectively.

### Factors associated with general anxiety symptoms severity

Only two participants had severe symptoms of anxiety, and therefore, they were grouped with those reporting moderate anxiety symptoms under one group (moderate to severe symptoms) in ordinal regression analyses. Table 2 presents unadjusted and adjusted associations between the characteristics of participants and general anxiety symptoms severity. In the unadjusted ordinal regression analyses, gender, nationality, and SARS-CoV-2 infection history were the only statistically significant variables associated with anxiety symptoms severity. In the adjusted ordinal regression analyses, gender and SARS-CoV-2 infection history were the only two variables showing statistically significant associations with general anxiety symptoms severity. Females had increased odds of higher levels of anxiety symptoms severity than males by 2.34 (95% CI 1.22, 4.50; p = 0.011). As compared to participants with unknown SARS-CoV-2 infection history, participants with a negative and positive SARS-CoV-2 infection history had increased odds of higher levels of anxiety symptoms severity by 2.48 (95% CI 1.17, 5.24; p = 0.017) and 7.21 (95% CI 1.67, 31.25; p = 0.008), respectively.

## Discussion

The present study assessed the prevalence of general anxiety symptoms severity and associated factors among a sample of older adults registered at one of the PHCC centers across Qatar during the early phase of the COVID-19 pandemic. Our study showed that only a small proportion of older adults had moderate to severe anxiety symptoms. Female gender and SARS-CoV-2 infection history were independently associated with higher anxiety symptoms. Age, marital status, living arrangements, nationality, and several medical conditions had no statistically significant associations with general anxiety symptoms severity.

The current study showed that most older adults had minimal to mild anxiety symptoms, and only 3.6% reported moderate to severe symptoms. This finding is consistent with previous population-based studies among older adults.^21–25^ For instance, the prevalence of moderate to severe anxiety symptoms during the COVID-19 pandemic was 2.4% and 0.5% among 2,032 Spanish older adults aged 60 years or older with or without a lifetime history of mental disorders, respectively.^22^ Another population-based survey across the United States during the COVID-19 pandemic showed that 6.3% of adults aged 65 years or older (n= 933) experienced moderate to severe anxiety symptoms.^24^ These findings suggest that older adults experienced a low prevalence of moderate to severe general anxiety symptoms during the early phase of the COVID-19 pandemic. This is consistent with the results of a systematic review identifying older age as a protective factor for mental health outcomes during the early phase of the COVID-19 pandemic as compared to pre-COVID-19 pandemic data.^9^ This observation could be explained by higher levels of resilience and more positive coping styles among older adults, which lead to better mental health outcomes.^26–29^ For example, a cross-sectional study among older adults under COVID-19 quarantine in Qatar showed that lower resilience scores were associated with higher severity of depression, anxiety, and stress symptoms.^29^ The relationship between effective adaptation with resilience and higher levels of mental well-being among older adults could be explained by diverse independent protective factors, including intrapersonal factors (such as perseverance and determination, self-efficacy and independence, a sense of purpose and meaning, and positive attitudes), spiritual factors (such as having faith and participating in prayer), interpersonal factors (such as sense of community and receiving social support from family, friends, neighbours, and health professionals), and experiential factors (such as learning from previous experiences, leading to more wisdom and proactive behaviours).^30^ Traditionally and religiously, older adults in Muslim and Arabic communities are treated with more respect and receive higher social support,^31^ which may enhance their resilience and psychological well-being. In the context of the COVID-19 pandemic and COVID-19-related fear, negative religious coping and lower levels of religiosity were independently associated with increased anxiety symptoms severity in previous studies from the Kingdom of Saudi Arabia (KSA) and Qatar.^32,33^

In the current study, the female gender was independently associated with increased severity of anxiety symptoms. This finding is also consistent with previous studies identifying female gender as a risk factor for more severe symptoms of anxiety and depression during the COVID-19 pandemic.^8,15^ It is generally well known that women have a greater risk of mental health conditions, such as anxiety and depression disorders, which could be explained by a complex interaction between various factors, including genetic, biological, and socioeconomic factors.^34,35^

In the present study, older adults with a positive or negative SARS-CoV-2 infection history had higher levels of anxiety symptoms severity than older adults with unknown SARS-CoV-2 infection status. This finding agrees with previous studies reporting more severe symptoms of anxiety and depression among individuals testing positive or negative (suspected cases) for SARS-CoV-2. A large population study (the COVID Symptom Study app) among 413,148 adults aged 18 years and older from the UK and the USA showed that self-reported SARS-CoV-positive was independently associated with increased odds of more severe anxiety and depression symptoms than SARS-CoV-negative among older adults aged 60 years and older.^36^ Another population-based study among 981 adults aged 18 years and older from the KSA, individuals who have been diagnosed with COVID-19 had higher odds of more severe anxiety symptoms than individuals who have not been diagnosed with COVID-19 by 5.5 times (95% CI 1.2, 24.0; P < 0.05).^37^ Higher levels of anxiety symptoms among older adults testing positive for SARS-CoV-2 could be explained by the fear of developing severe COVID-19 infection and the risk of death.^38,39^ Similarly, increased levels of anxiety symptoms among those testing negative for SARS-CoV-2 may be explained by the fear of being infected, especially if they were in contact with confirmed or probable cases of COVID-19.^40,41^ For example, a population-based study among individuals aged 18 to 75 years from the KSA found that individuals who reported contact with COVID-19 cases during the last month had higher anxiety symptoms severity than individuals reporting no contact with COVID-19 cases (42.9% vs. 15.8%, respectively).^41^ Similarly, the same study found that previously quarantined individuals had higher anxiety symptoms severity than individuals who were not quarantined (31.6% vs. 16%, respectively).

The present study has several strengths. First, this study used a random sample of older adults from all PHCC centres in Qatar, which is representative of older adults registered with the PHCC centres in terms of gender and nationality. Second, the majority (88%) of the randomly selected and contacted individuals participated in the study. Third, the anxiety symptoms severity was measured using the GAD, which is a validated scale. Fourth, health-related variables and SARS-CoV-2 infection history were extracted from medical records, which minimizes self-reporting and recall biases. Fifth, data collection and phone interviews with participants were conducted by family physicians following a standardized interview script.

This study also has some limitations that should be acknowledged. First, the severity of anxiety symptoms was assessed using phone interviews due to the physical distancing policies implemented in Qatar during the early stage of the COVID-19 pandemic and the fact that some older adults may be unable to complete an online survey. Therefore, some responses to the GAD-7 scale questions may have introduced some social desirability bias due to the stigma attached to expressing negative emotions in the Arab population,^42^ which may underestimate the prevalence of anxiety symptoms severity and the magnitude of observed associations. Second, this study collected information on key sociodemographic and health-related characteristics but did not account for other factors associated with anxiety symptoms, such as socioeconomic status, the severity of their health conditions, and personal relationships.^15^ Third, our findings may not be generalized to older adults not registered with the PHCC centres during the study period. Fourth, this was a cross-sectional study (a snapshot at a given time). Therefore, the direction of observed associations between SARS-CoV-2 infection history and anxiety symptoms severity remains unclear because we are unsure if the observed anxiety symptoms severity existed before SARS-CoV-2 infection history.

Despite these limitations, our study indicates that older adults, females, and those with confirmed or suspected SARS-CoV-2 infection experienced more severe anxiety symptoms. Screening and evaluating this vulnerable group of older adults for general anxiety disorder and other comorbid mental disorders and targeting them with tailored psychological support interventions may reduce their anxiety levels and any related negative impact on physical and mental health. For example, there is some evidence that targeted and remotely delivered psychological support interventions are effective in reducing levels of anxiety and depression among those exposed to mass communicable disease outbreaks, including COVID-19.^43^ Additionally, recent systematic reviews of randomized controlled trials showed that telemedicine or online mindfulness-based psychological interventions during the COVID-19 pandemic were associated with beneficial effects on anxiety, depression, and psychological distress symptoms.^44,45^

## Conclusion

Most older adults registered with the PHCC in Qatar reported minimal to mild anxiety symptoms during the initial phase of the COVID-19 pandemic. However, females and individuals with confirmed or suspected SARS-CoV-2 infection had higher levels of anxiety symptoms severity. More longitudinal research is needed to better understand the long-term impact of the COVID-19 pandemic on mental health among older adults and associated factors. Our findings are consistent with previous studies suggesting that most older adults may be resilient to COVID-19-related anxiety. Therefore, more research is needed to better understand the factors and mechanisms underlying psychological resilience among older adults. This information can be especially useful in developing targeted interventions to prevent or reduce the impact of COVID-19-related anxiety among older adults affected by COVID-19 and other high-risk groups.

## Figures and Tables

**Table 1.** Characteristics of participants.

**Variable**	**Frequency (%)**
Age (years)	65.0 (4.8)*
Gender	
Female	165 (49.0)
Male	172 (51.0)
Nationality	
Qatari	108 (32.0)
Other	229 (68.0)
Living arrangements	
Lives with family	301 (89.3)
Lives alone	36 (10.7)
Marital status	
Married	313 (92.9)
Not Married	24 (7.1)
COVID-19 infection status	
Unknown	113 (33.5)
Negative	214 (63.5)
Positive	10 (3.0)
Diabetes	
No	144 (42.7)
Yes	193 (57.3)
Hypertension	
No	138 (41.0)
Yes	199 (59.0)
Cardiovascular disease	
No	286 (84.9)
Yes	51 (15.1)
Dyslipidemia	
No	170 (50.4)
Yes	167 (49.6)
Asthma/chronic obstructive pulmonary disease	
No	290 (86.0)
Yes	47 (14.0)
Cerebrovascular Disease	
No	331 (98.2)
Yes	6 (1.8)
Cancer	
No	314 (93.2)
Yes	23 (6.8)
Chronic kidney disease	
No	314 (93.2)
Yes	23 (6.8)
Liver Disease	
No	331 (98.2)
Yes	6 (1.8)

* Mean (standard deviation)

**Table 2.** Crude and adjusted associations between sociodemographic and medical characteristics and general anxiety symptoms severity.

	**General anxiety symptoms’ severity**			**Unadjusted association**		**Adjusted association**	
**Variable**	**Minimal Frequency (%)**	**Mild Frequency (%)**	**Moderate to severe Frequency (%)**	**OR (95% CI)**	**p-value**	**OR (95% CI)**	**p-value**
Age (years)	278 (64.9, 4.6)*	47 (65.9, 6.0)*	12 (63.9, 4.1)*	1.02 (0.97, 1.08)	0.425	1.01 (0.95, 1.07)	0.708
Gender							
Male	153 (89.0)	14 (8.1)	5 (2.9)	1.00		1.00	
Female	125 (75.8)	33 (20.0)	7 (4.2)	2.52 (1.39, 4.57)	0.002	2.34 (1.22, 4.50)	0.011
Nationality							
Other	196 (85.6)	25 (10.9)	8 (3.5)	1.00		1.00	
Qatari	82 (75.9)	22 (20.4)	4 (3.7)	1.83 (1.04, 3.25)	0.038	1.30 (0.67, 2.50)	0.443
Living arrangements							
Lives with family	247 (82.1)	43 (14.3)	11 (3.7)	1.00		1.00	
Lives alone	31 (86.1)	4 (11.1)	1 (2.8)	0.74 (0.28, 1.98)	0.547	0.82 (0.28, 2.42)	0.717
Marital status							
Not Married	18 (75.0)	3 (12.5)	3 (12.5)	1.00		1.00	
Married	260 (83.1)	44 (14.1)	9 (2.9)	0.55 (0.21, 1.46)	0.231	0.50 (0.17, 1.46)	0.204
COVID-19 status							
Unknown	103 (91.2)	7 (6.2)	3 (2.7)	1.00		1.00	
Negative	169 (79.0)	37 (17.3)	8 (3.7)	2.69 (1.30, 5.57)	0.008	2.48 (1.17, 5.24)	0.017
Positive	6 (60.0)	3 (30.0)	1 (10.0)	6.78 (1.68, 27.38)	0.007	7.21 (1.67, 31.25)	0.008
Number of comorbidities							
0	52 (80.0)	12 (18.5)	1 (1.5)	1.00		1.00	
1	48 (85.7)	7 (12.5)	1 (1.8)	0.68 (0.26, 1.78)	0.431	0.83 (0.30, 2.26)	0.712
2	60 (84.5)	7 (9.9)	4 (5.6)	0.78 (0.32, 1.89)	0.582	0.63 (0.25, 1.63)	0.344
3	74 (84.1)	13 (14.8)	1 (1.1)	0.76 (0.33, 1.75)	0.522	0.53 (0.21, 1.34)	0.180
≥4	44 (77.2)	8 (14.0)	5 (8.8)	1.28 (0.54, 3.05)	0.570	0.99 (0.36, 2.69)	0.983

Abbreviations: OR, odds ratio; CI, confidence interval.

Notes: *Numbers are mean (standard deviation). Model summary for multivariable ordinal logistic regression: Log-likelihood = -173.60372; Chi-Square (11) = 25.02, p= 0.009; Pseudo R-Squared = 0.067.

## Appendix

**APPENDIX A:** GENERALIZED ANXIETY DISORDER 7-ITEM (GAD-7) SCALE*

**Over the last two weeks, how often have you been bothered by the following problems?**	**Not at all sure**	**Several days**	**Over half the days**	**Nearly every day**
1. Feeling nervous, anxious, or on edge.	0	1	2	3
2. Not being able to stop or control worrying.	0	1	2	3
3. Worrying too much about different things.	0	1	2	3
4. Trouble relaxing.	0	1	2	3
5. Being so restless that it’s hard to sit still.	0	1	2	3
6. Becoming easily annoyed or irritable.	0	1	2	3
7. Feeling afraid as if something awful might happen.	0	1	2	3
**Add the score for each column.**				

**Total Score (add your column scores) =**

If you checked off any problems, how difficult have these made it for you to do your work, take care of things at home, or get along with other people?

□ Not difficult at all

□ Somewhat difficult

□ Very difficult

□ Extremely difficult

* Developed by Drs. Robert L. Spitzer, Janet B.W. Williams, Kurt Kroenke, and colleagues, with an educational grant from Pfizer Inc. No permission required to reproduce, translate, display, or distribute, 1999.
